# Influenza A Virus Inhibits RSV Infection via a Two-Wave Expression of IFIT Proteins

**DOI:** 10.3390/v12101171

**Published:** 2020-10-16

**Authors:** Yaron Drori, Jasmine Jacob-Hirsch, Rakefet Pando, Aharona Glatman-Freedman, Nehemya Friedman, Ella Mendelson, Michal Mandelboim

**Affiliations:** 1 Central Virology Laboratory, Ministry of Health, Chaim Sheba Medical Center, Ramat Gan 5265601, Israel; Yaron.Drori@sheba.health.gov.il (Y.D.); Rakefet.Pando@sheba.health.gov.il (R.P.); Nehemya.Friedman@sheba.health.gov.il (N.F.); Ella.Mendelson@sheba.health.gov.il (E.M.); 2 Department of Epidemiology and Preventive Medicine, School of Public Health, Sackler Faculty of Medicine, Tel-Aviv University, Tel-Aviv 69978, Israel; Aharona.Freedman@MOH.GOV.IL; 3 Functional Genomics Unit, Institute of Hematology, Sheba Medical Center, Tel Hashomer, Ramat Gan 5265601, Israel; Jasmine.Jacob@sheba.health.gov.il; 4 Chaim Sheba Medical Center, Israel Center for Disease Control, Ministry of Health, Ramat Gan 5265601, Israel

**Keywords:** Influenza A, RSV, IFITs, double infection

## Abstract

Influenza viruses and respiratory syncytial virus (RSV) are respiratory viruses that primarily circulate worldwide during the autumn and winter seasons. Seasonal surveillance has shown that RSV infection generally precedes influenza. However, in the last four winter seasons (2016–2020) an overlap of the morbidity peaks of both viruses was observed in Israel, and was paralleled by significantly lower RSV infection rates. To investigate whether the influenza A virus inhibits RSV, human cervical carcinoma (HEp2) cells or mice were co-infected with influenza A and RSV. Influenza A inhibited RSV growth, both in vitro and in vivo. Mass spectrometry analysis of mouse lungs infected with influenza A identified a two-wave pattern of protein expression upregulation, which included members of the interferon-induced protein with the tetratricopeptide (IFITs) family. Interestingly, in the second wave, influenza A viruses were no longer detectable in mouse lungs. In addition, knockdown and overexpression of IFITs in HEp2 cells affected RSV multiplicity. In conclusion, influenza A infection inhibits RSV infectivity via upregulation of IFIT proteins in a two-wave modality. Understanding the immune system involvement in the interaction between influenza A and RSV viruses will contribute to the development of future treatment strategies against these viruses.

## 1. Introduction

Respiratory syncytial virus (RSV) and influenza viruses are two respiratory viruses associated with significant morbidity and mortality worldwide [1]. RSV is a leading pathogen causing acute lower respiratory tract infection (ALRTI) [2] in all age groups [3,4], primarily infants and young toddlers [5,6], while the influenza virus affects all age groups [7]. In Israel, RSV is usually detected in October, with a clear peak in December, whereas the influenza virus arrives in November and peaks in January [8]. Previous surveillance demonstrated a close temporal relationship between circulating influenza viruses and RSV, with influenza epidemics usually occurring when RSV infections subsided [9]. However, in the last four seasons, influenza infection overlapped with that of RSV in Israel, which resulted in decreased RSV-related morbidity.

The activity of both innate and adaptive immune systems against both viruses is very well documented [2,10]. Innate immunity against these viruses includes, among others, the interferon-stimulating gene (ISG) pathway, which triggers the expression of the interferon-induced protein with the tetratricopeptide (IFIT) family of proteins [11,12]. In humans, IFIT1–3 proteins form a complex with each other to inhibit translation of viral mRNA molecules [13]. IFIT1 acts as a sensor that identifies specific viral single-stranded RNAs and selectively inhibits viral protein synthesis without affecting host cell protein synthesis [14]. While IFIT1 is known to specifically recognize the viral mRNA [14], it has been suggested that IFIT2 and IFIT3 facilitate the binding of the IFIT1:2:3 complex structure to the viral mRNA [15,16]. IFI44 is also an ISG [17] and its expression is induced following infection with different viruses [18,19]. However, less is known about IFI44 function against these viruses. In influenza virus-infected cells, IFI44 is upregulated and was found to regulate the innate immune responses by negatively modulating interferon response pathways to control exacerbated immune reaction [20]. The present work investigated the role of IFIT1–3 and IFI44 proteins in the cross-immunity effect observed between influenza and RSV.

## 2. Materials and Methods

### 2.1. Clinical Samples and Ethical Approval

Nasopharyngeal samples were collected from both hospitalized and non-hospitalized patients presenting influenza-like illness (ILI) symptoms. Samples from non-hospitalized patients with ILI were collected as part of the seasonal influenza surveillance network, which operates in collaboration with the Israeli Center for Disease Control (ICDC) in dozens of clinics in Israel (Helsinki Number 1967-15-SMC). Samples from hospitalized ILI patients were collected at the Sheba Medical Center ((Helsinki Number 1969-15-SMC). Informed consent was not required for this study. This retrospective analysis included the samples obtained from hospitalized patients and was conducted for samples collected in the years 2013–2014 (*n* = 5098), 2014–2015 (*n* = 4653), 2015–2016 (*n* = 4982), 2016–2017 (*n* = 6098), 2017–2018 (*n* = 6579), 2018–2019 (*n* = 7280), and 2019–2020 (*n* = 7794), and samples collected from outpatients during the same period (2013–2014, *n* = 1755; 2014–2015, *n* = 1142; 2015–2016, *n* = 1919; 2016–2017, *n* = 1284; 2017–2018, *n* = 1461; 2018–2019, *n* = 1488; and 2019–2020, *n* = 2051). Samples included were collected between October and April of each year.

### 2.2. Viral RNA Extraction

Viral RNA was extracted from cell supernatants using the MagNA PURE (ROCHE, Copenhagen, Denmark) 96 instrument, according to the manufacturer’s instructions. Briefly, nucleic acids were extracted from 500 µL of each sample and eluted in 50 µL elution buffer. Quantitative real-time polymerase chain reactions (qPCR) were performed using the Ambion Ag-Path master mix (Life Technologies, Austin, TX 78744, USA) and the ABI 7500 instrument, to test for the presence of influenza viruses, as previously described [8,21] Determination of human RSV was performed using qPCR, as previously described [8].

### 2.3. Co-Infection of HEp2 Cells with Influenza A/H3N2 and RSV

Human cervical carcinoma (HEp2; ATCC) cells (1 × 10^6^), which are susceptible to both influenza virus and RSV, were grown in 6-well plates (Nunc^TM^) in 2 mL 10% fetal calf serum (FCS)-enriched MEM-Eagle, Earle’s salts (Biological Industries, Kibbutz Beit-Haemek, Israel), and incubated overnight (37 °C, 5% CO_2_). The cells were washed with phosphate buffered solution (PBS) and infected with 6 × 10^5^ PFU (Multiplicity of infection [MOI] = 0.1) influenza A/H3N2 (A/Israel/C9037/2017, GISAID ID EPI_ISL_297343). Infections were performed in the presence of 1 μg/mL tosylsulfonyl phenylalanyl chloromethyl ketone (TPCK)-treated trypsin (Sigma-Aldrich, Rehovot, Israel). After 3 h, the cells were washed with PBS and then infected with 6 × 10^5^ PFU of RSV (ATCC® VR-1540). In separate 6-well plates, 1 × 10^6^ HEp2 cells were grown in 2 mL 10% FCS-enriched MEM-Eagle, Earle’s salts (Biological Industries, Israel), and incubated overnight (37 °C, 5% CO_2_). The cells were then washed with PBS and infected with RSV (6 × 10^5^ PFU, MOI = 0.1). After 3 h, the cells were washed with PBS, and then infected with influenza A/H3N2 (6 × 10^5^ PFU, MOI = 0.1). Following washing with PBS, the cells were incubated (37 °C, 5% CO_2_) in 3 mL 2% FCS-enriched MEM-Eagle, Earle’s salts. Supernatant was collected at predefined time points (24, 48, 72, 96 and 144 h) and RNA was extracted. qPCR was performed to quantify the level of the virus at each time point.

### 2.4. Co-Infection of Mice with Influenza A/PR/8/1934 and RSV

*Balb/c* mice (2.5-week-old) were anesthetized with isoflurane (Piramal Critical Care, PA 18017, USA) inhalation liquid and intranasally infected with influenza A/PR/8/1934 (“influenza A”) (4 × 10^2^ PFU/mL). The mice were split into groups according to the days post influenza A/PR/8/1934 infection (1, 2, 3, 5, 6, 8, 10, and 12); three mice from each group were subsequently infected with RSV (6 × 10^6^ PFU/mL) simultaneously for 4 days then sacrificed. The presence of the viruses was tested by grinding mouse lungs in 1ml DMEM (Biological Industries, Kibbutz Beit-Haemek, Israel) using the Spex centri prep 8000-D Mixer Mill homogenizer (BENCHMARK SCIENTIFIC, NJ 08818, USA), for 10 min, followed by RNA extraction and qPCR, as described [8], and was compared to the lungs of control mice (infected with RSV only).

### 2.5. Mass Spectrometry

Infected mouse lung samples were homogenized in 1 mL DMEM (Biological Industries, Kibbutz Beit-Haemek, Israel) with protease inhibitor “cOmplete“ ULTRA Tablets, Mini, EASYpack (ROCH), 1 tab/1 mL DMEM, and analyzed by mass spectrometry at the Smoler Proteomics Center, Technion, Haifa Israel [22].

### 2.6. Genes Silencing of IFIT1, IFIT2, IFIT3 and IFI44

We generated stable cell lines in which IFIT1–3 and IFI44 proteins were silenced by using lentiviral particles of *sh*RNA specific for human IFIT1, IFIT2, IFIT3, and IFI44, as well as non-targeting *sh*RNA. Lentiviral particles were purchased from Sigma-Aldrich, Rehovot, Israel. Transduction into HEp2 cells was performed according to the manufacturer’s instructions. HEp2 clones were selected with puromycin 6µg/mL (Sigma-Aldrich, Rehovot, Israel), and viral RNA was detected by reverse transcriptase-polymerase chain reaction (RT-PCR) performed using the following primers:

IFIT1
forward, 5′-TCTCAGAGGAGCCTGGCTAA-3′
reverse, 5′-TCAGGCATTTCATCGTCATC-3′
IFIT2
forward, 5′-CGAACAGCTGAGAATTGCAC-3′
reverse, 5′-TGCACATTGTGGCTTTGAAT-3′
IFIT3
forward, 5′-CGGAACAGCAGAGACACAGA-3′
reverse, 5′-CTGCCTCGTTGTTACCATCT-3’
IFI44
forward, 5′- AGCTGGGAAGTCCAGCTTTT-3′
reverse, 5′-CCCCAGTGAGTCACACAGAA-3′

The silenced clones were named: “HEp2 *shIFIT1*, HEp2 *shIFIT2*, HEp2 *shIFIT3*, and HEp2*shIFI44*”.

### 2.7. Gene Overexpression of IFIT1, IFIT2, IFIT3, and IFI44

We generated stable cell lines overexpressing IFIT1–3 and IFI44 proteins. Lentiviral vectors were produced in 293T cells using the TransIT-LT1 transfection reagent (Mirus, ZOTAL, Tel-Aviv, Israel) in a transient three-plasmid transfection protocol according to the manufacturer’s instructions. The expression vector used was pHAGE-DsRED(−) eGFP(+). Primers designed to create the insert were as follows:

IFIT1
forward, 5′-TTTTCTCGAGGCCGCCACCATGAGTACAAATGGTGATGATC-3′
reverse, 5′-AAAGCTAGCCTAAGGACCTTGTCTCACAGAGTTC-3′
IFIT2
forward, 5′-TTTTCTCGAGGCCGCCACCATGAGTGAGAACAATAAGAA-3′
reverse, 5′-AAAGCTAGCTCATTCCCCATTCCAGCTTGATGCT-3′
IFIT3
forward, 5′-TTTTCTCGAGGCCGCCACCATGAGTGAGGTCACCAAGAATTC-3′
reverse, 5′-AAAGCTAGCTCAGTTCAGTTGCTCTGAGTTAG-3′
IFI44
forward, 5′-TTTTCTCGAGGCCGCCACCATGGCAGTGACAACTCGTTTGAC-3′
reverse, 5′-CCCGCTAGCCTACCCGCTAGCCTATTTTTTTCCTTGTGCACAGTTGATAATTTCCTCCCTTAGATTC-3′

The overexpression clones were named: “IFIT1 O/E, IFIT2 O/E, IFIT3 O/E, and IFI44 O/E”.

### 2.8. Western Blot

Whole-cell extracts were eluted using Pierce^TM^ RIPA buffer (Thermo SCIENTIFIC, IL 61101, USA), according to the manufacturer’s protocols, fractionated by SDS-PAGE 4–20% (Mini-PROTEAN TGX Stain-free Gels, BIO-RAD, USA) and transferred to a 0.2 µM nitrocellulose membrane (Trans-Blot Turbo Transfer Pack, BIO-RAD, USA) using a Turbo® Transfer System, according to the manufacturer’s protocols (BIO-RAD, USA). After incubation with InstantBlock buffer (Gene Bio-Application L.T.D, Yavne, Israel), according to the manufacturer’s protocol, the membrane was washed once with Tris buffered saline with Tween 20 (TBST) (10 mM Tris, pH 8.0, 150 mM NaCl, 1% Tween 20) and incubated with polyclonal antibodies (Thermo SCIENTIFIC, IL 61101, USA) specific for IFIT1 (1:1000), IFIT2 (1:1000), IFIT3 (1:1000), IFI44 (1:1000) or GAPDH (1:5000), for 1–3h, at 4 °C. Membranes were washed three times and incubated with a 1:10,000 dilution of horseradish peroxidase-conjugated anti-rabbit antibodies (Sigma-Aldrich, Rehovot, Israel) for 1–2 h, at room temperature. Blots were washed with TBST and developed with the Clarity^TM^ Western ECL substrate (BIO-RAD, USA), according to the manufacturer’s protocols.

### 2.9. Infection of IFIT-Silenced/Overexpressed HEp2 Cells with RSV

IFIT-silenced/overexpressing HEp2 (1 × 10^6^) cells were grown overnight in 6-well plates (NuncTM) in 10% FCS-enriched EMEM (37 °C, 5% CO2). The cells were washed with 1 mL PBS and infected with RSV (6 × 10^5^ PFU, MOI = 0.1). After 3 h, the cells were washed with 1 mL PBS, and incubated in 2% FCS-enriched MEM-Eagle, Earle’s salts (37 °C, 5% CO_2_), for 5 days. Supernatant was collected and subjected to qPCR, as previously described [8], to test for the presence of human RSV.

### 2.10. Reverse Transcriptase-Polymerase Chain Reaction (RT-PCR)

RNA samples were subjected to RT-PCR, performed with the SuperScript III One-Step RT-PCR system (Invitrogen, Carlsbad, CA, USA), according to the manufacturer’s instruction. Briefly, the reaction mixture contained 200 ng sample RNA, 5 pmoles of each forward and reverse PCR primer, and the SuperScript III reverse transcriptase/Platinum taq polymerase mixture provided by the manufacturer, in 25 µL of the recommended buffer. The reaction consisted of incubation at 55 °C for 30 min for reverse transcription, followed by 35 cycles of 94 °C for 20 s, 58 °C for 30 s, and 68 °C for 30 s. Five microliters of the amplified reaction was electrophoresed on a 2% agarose gel, followed by staining with ethidium bromide. Expression data were normalized to the geometric mean of housekeeping gene GAPDH to control the variability in expression levels and were analyzed using ImageJ v1.47 (National institute of health, USA). Primers used were as follows:

Mice lung:
Ifit1
forward, 5′-TGCTGAGATGGACTGTGAGG-3′
reverse, 5′-GTGCCAATTCTTGCACATTG-3′
ifit2
forward, 5′-ACATGGGCCAGTTCTCAAAG-3′
reverse, 5′-ATGCACATAGGCGTTGTTTG-3′
ifit3
forward, 5′-CTGGTCACCTGGGGAAACTA-3′
reverse, 5′-ATCGCAGTGCTTTTCCAAGT-3′
ifi44
forward, 5′-GTTTGACATGGCAGCAAGAA-3′
reverse, 5′-TATTGCGAGCAGTCAGTTGG-3′

### 2.11. Statistical Analysis

One-way ANOVA analysis was performed to evaluate the differences in percent positivity between the compared groups. Values were considered statistically significant at *p* < 0.05. All analyses were performed using IBM® SPSS® Statistics software (Version 23) and Excel software (Microsoft®).

## 3. Results

### 3.1. Influenza Infection Inhibits RSV

**Figure 1:**
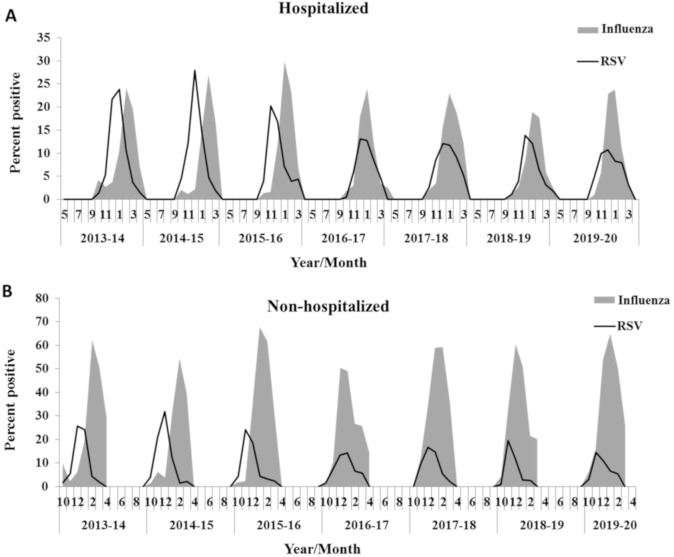
An overview of the percentage of influenza-positive versus RSV-positive samples in winter seasons 2013–2020. (**A**) The monthly percentage of positive cases of infection with respiratory viruses (RSV, and/or influenza) from 2013 to 2020 among hospitalized patients. (**B**) The monthly percentage of positive cases of infection with respiratory viruses (RSV, and/or influenza) from 2013 to 2020 among non-hospitalized patients. The black line represents the infection patterns for RSV and the gray area represents influenza infection patterns. The percentage of patients bearing each virus was calculated each month in relation to the total number of samples tested for the presence of that virus. RSV’s peaks from 2016–2020 were compared to those from 2013–2016 in hospitalized/non-hospitalized patients (*p* < 0.001 one-way ANOVA followed by post-hoc Bonferroni *t*-test).

In the winter seasons 2013–2016, RSV infection was detected in October, and peaked in December, while influenza infections peaked in the middle of January (Figure 1A) and February (Figure 1B). The dominant influenza strain in winter seasons 2013–2014 and 2013–2015 was influenza A/H3N2 (46.9% and 85% positive cases, respectively), while in winter season 2015–2016, the dominant strain was influenza B (56.4% positive cases). In 2016–2017 the dominant influenza strain was influenza A/H3N2 (97.7% positive cases), while in 2017–18, the dominant strain was influenza B (70% positive cases). In 2018–19, influenza A/H3N2 was the dominant strain (76.5% positive cases) and in 2019–2020, influenza A/H1N1pdm09 was the dominant strain with 61.86% positive cases (Figure 1A,B). Surprisingly, from the winter of 2016–2017 and onwards, both RSV and influenza co-circulated in October and both peaked around January (Figure 1A,B). This early circulation of influenza in the winters of 2016–2020 coincided with a lower annual incidence of RSV as compared to previous seasons (2013–2016), and also coincided with later peaks of RSV in January (*p* < 0.001). The co-infection of both viruses was observed both in hospitalized and in non-hospitalized patients (Figure 1A,B, respectively). Overall, it seems as if influenza infection inhibits RSV infection, regardless of the identity of the dominant influenza strain.

### 3.2. Influenza Infection Inhibits RSV In Vitro

**Figure 2:**
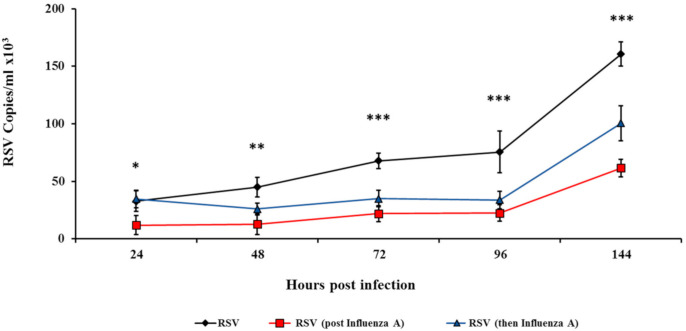
Co-infection of HEp2 cells with influenza A/H3N2 and RSV. HEp2 cells (1 × 10^6^) were infected with influenza A/H3N2 (6 × 10^5^ PFU) for 3 h, and then with RSV (6 × 10^5^ PFU), and vice versa. RSV-infected cells served as control. RNA was extracted from supernatant samples collected at predefined time points (24–144 h). Quantitative real-time polymerase chain reaction (qPCR) was performed to test for viral quantity. The black line indicates infection with RSV, the red line indicates infection with influenza A/H3N2 followed by RSV, and the blue line indicates infection with RSV followed by influenza A/H3N2. The data presented are an average of three independent experiments ± mean standard deviation * *p* < 0.05, ** *p* < 0.01 and *** *p* < 0.001.

To test whether influenza virus can inhibit RSV, HEp2 cells were infected with influenza A virus for 3 h, followed by RSV infection, and vice versa. The number of RSV viral copies was then determined at various time points after the second infection. When applied alone, RSV copy numbers increased with time (24, 48, 72, 96, and 144 h). However, when cells were first infected with influenza A and then RSV, or vice versa, significantly fewer RSV copies were observed 24–144 h after the second infection (Figure 2, *p* < 0.05). Similar results were obtained when HEp2 cells were infected with influenza A virus for 12 h, followed by RSV infection, and vice versa significantly fewer RSV copies were observed 48–144 h after the second infection (Supplementary Figure S1, *p* < 0.05).

### 3.3. Influenza Infection Inhibits RSV In Vivo in a Two-Wave Modality

**Figure 3:**
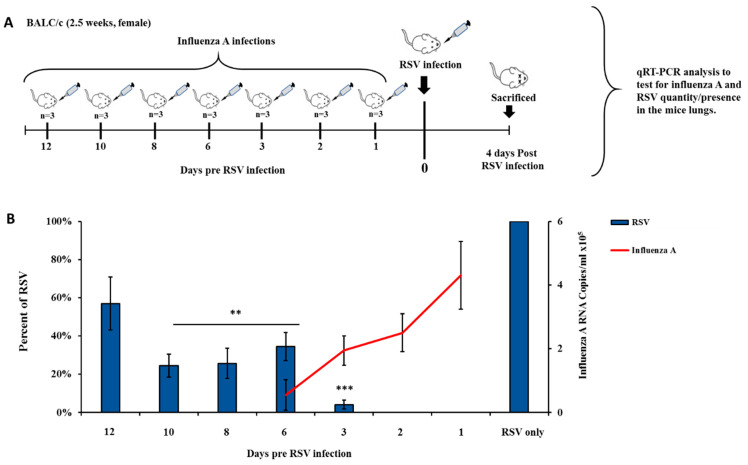
Viral loads in mouse lungs co-infected with influenza A (A/PR/8/1934) and RSV. (**A**) Experimental design scheme. Balb/c mice (2.5 weeks old) were intranasally infected with influenza A/PR/8/1934 (4 × 10^2^ PFU/mL) at predefined time points (1, 2, 3, 5, 6, 8, 10, and 12 days) pre-RSV infection (6 × 10^6^ PFU/mL). Mice were sacrificed four days after RSV infection. Mice that were infected with RSV only were sacrificed four days later and served as the control group. qPCR analysis was performed to test for viral quantity. (**B**) Mouse lungs were homogenized using the Spex centri prep 8000-D Mixer (Mill). RNA was then extracted and the amount of virus was determined by qPCR. Left Y axis presents RSV percent compared to control group (blue columns), right Y axis presents influenza A/PR/8/1934 RNA copies/mL (Red line). The data presented are an average of three independent experiments ± mean standard deviation. * *p* < 0.05, ** *p* < 0.01 and *** *p* < 0.001.

To test whether influenza infection inhibits RSV proliferation in vivo, various groups of mice (3 in each group), were infected with influenza A/PR8/1934 virus 12, 10, 8, 6, 3, 2, or 1 days subsequent to RSV infection. All mice groups were next infected with RSV (designated as day 0 in Figure 3A) and four days after RSV infection, mice were sacrificed to check the quantity/presence of RSV and influenza A/PR8/1934 virus in the lung. (Figure 3A). Mice infected with RSV only were sacrificed four days after infection and served as the control group (Figure 3A). The presence of influenza A virus and RSV was examined in the mice lungs (Figure 3B). In mice that had been infected with influenza A 1–2 days subsequent to RSV infection, RSV was not detected at all. RSV was detected in mice infected with influenza A, 3 or more days subsequent to RSV infection (Figure 3B). Hence, a first wave of RSV inhibition occurred between day 1 and day 3 pre-RSV infection. From day 3 pre-RSV infection, in parallel to the decline in influenza A virus levels, RSV levels increased in the lungs of the mice, reaching a peak on day 6 pre-RSV infection (Figure 3B). A second wave of RSV inhibition was observed from day 8 until day 10 pre-RSV infection, despite the absence of detectable influenza virus in the lungs at these time points (Figure 3B).

### 3.4. Two-Wave Elevation of Anti-Viral Proteins Following Influenza Infection

**Figure 4:**
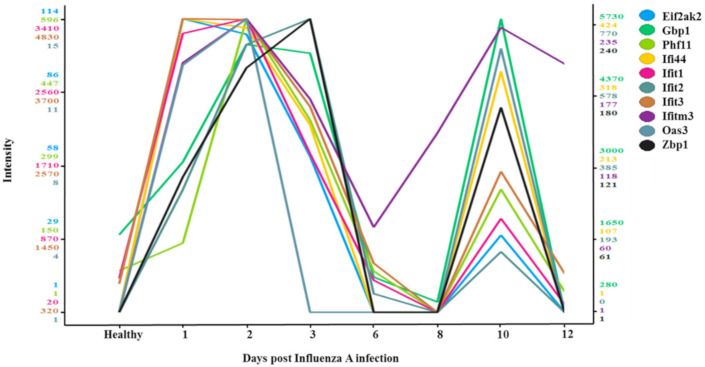
Mass spectrometry analyses of influenza-infected mouse lungs (A/PR/8/1934) versus healthy (non-infected) mouse lungs. Plot of antiviral gene expression levels in influenza A/PR/8/1934-infected cells (2-fold change influenza A/PR/8/1934 vs. healthy) with expression patterns similar to that of IFIT1 (correlation ≥ 0.6, Tibco Spotfire V.7.7.0. software). The color scale (*y* axis) for each gene is independent, based on the distribution of the protein’s expression levels across the samples. The samples plotted (*x* axis) are the healthy, non-infected cells and the post-influenza A/PR/8/1934 infection samples, collected on day 1–3, day 6, day 8, day 10, and day 12.

**Figure 5:**
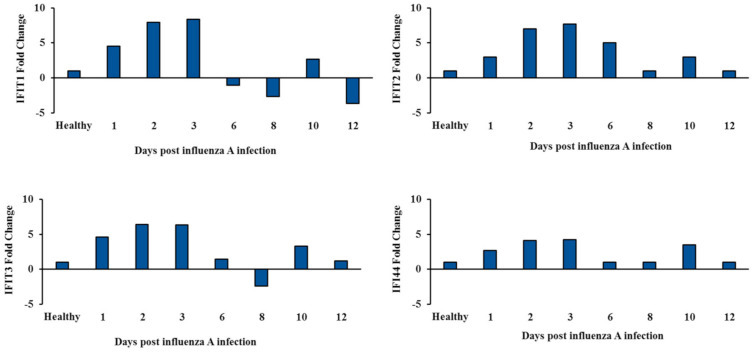
IFIT1–3 and IFI44 protein expression pattern. Mass spectrometry analyses of influenza-infected mice lungs (A/PR/8/1934) versus healthy (non-infected). The data presented are an average fold change of each protein normalized to an average fold change of the protein in the healthy mice.

**Figure 6:**
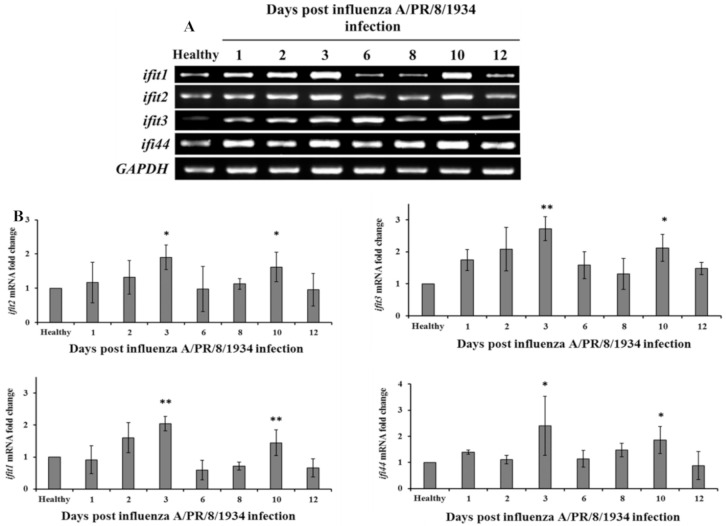
*IFIT1–3* and *IFI44* mRNAs expression pattern. (**A**) Agarose gel of IFIT1–3 and IFI44 mRNAs of healthy mice and mice infected with influenza A. Balb/c mice (2.5 weeks old) were intranasally infected with influenza A/PR/8/1934 (4 × 10^2^ PFU/mL). Mice were sacrificed at various time points (1, 2, 3, 5, 6, 8, 10, and 12 days) post-infection. Non-infected mice served as the control group. Mouse lungs were homogenized using the Spex centri prep 8000-D Mixer (Mill). RNA was then extracted. Reverse transcriptase-polymerase chain reaction (RT-PCR) was performed to test for IFIT1–3 or IFI44 mRNAs levels. (**B**) Relative quantification of IFIT1–3 and IFI44 mRNAs levels were normalized to the geometric mean of housekeeping gene GAPDH. The data presented are an average of three independent experiments ± mean standard deviation. The data presented is an average of three independent experiments ± mean standard deviation. * *p* < 0.05, ** *p* < 0.01 and *** *p* < 0.001.

To understand the two-wave effect of influenza on RSV, a mass spectrometry analysis was performed on samples from healthy mice and from mice infected with influenza A. A total of 10 proteins demonstrated a two-wave upregulation pattern (Figure 4); all were proteins known to have antiviral activity (Table 1). Specific focus was placed on four out the 10 antiviral proteins; all four belonged to the IFIT family (IFIT1, IFIT2, IFIT3, and IFI44) and exhibit antiviral functions specifically against RNA viruses such as the RSV. The four proteins were upregulated on days 1–3 post-infection and then again on day 10 post-infection (Figure 5). To test whether the upregulation of IFIT expression observed in the second wave could be due to new mRNAs synthesis or due to other reasons, such as post-transcription modifications, RT-PCR was performed on RNA acquired at different time points from the infected mouse lungs (Figure 6A). Expression of all four IFIT mRNAs increased by up to 2.5–fold on days 1–3 post-infection, decreased on days 6–8, and increased by up to 1.5–fold once again on day 10 post-infection (Figure 6B).

### 3.5. IFIT Members Inhibit RSV Infection

**Figure 7:**
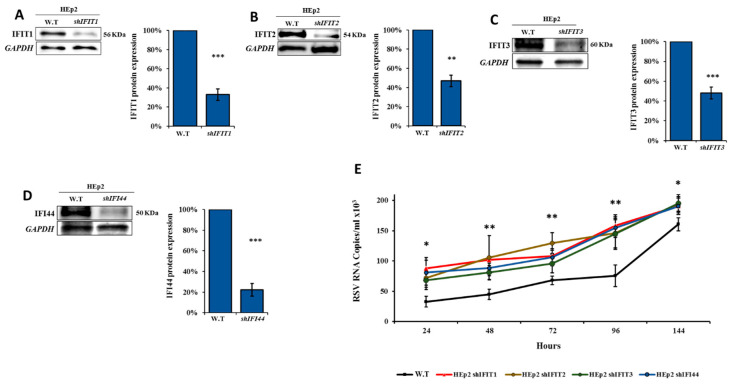
Infection of IFIT1–3- and IFI44-silenced HEp2 cells with RSV. (**A**–**D**) Lentiviral vectors carrying *sh*RNA targeting *IFIT1*, *IFIT2*, *IFIT3*, or *IFI44* were infected into HEp2 cells. Following selection by puromycin, whole-cell extracts were harvested after 5 days of infection. Western blot analysis using antibodies specific for (a) IFIT1, (b) IFIT2, (c) IFIT3, or (d) IFI44. The bars on the graph present the quantification of IFIT1–3, IFIT4, IFIT1–3, and IFI44 expression levels (32.97%, 46.88%, 53.37%, and 34.20%, respectively) as compared to their levels in untreated control cells. Error bars represent S.E.* *p* < 0.05, ** *p* < 0.01 and *** *p* < 0.001. (**E**) shIFIT1/shIFIT2/shIFIT3/shIFI44-silenced HEp2 cells (1 × 10^6^) were infected with RSV (6 × 10^5^ PFU, 3 h). Supernatant samples were collected at predefined time points (24–144 h) post infection and RNA was extracted. The control growth curve (black line) is RSV-infected HEp2 cells (W.T) and is the same data represented in Figure 2. qPCR analysis was performed to test for viral quantity. The data presented are an average of the independent experiments ± mean standard deviation. * *p* < 0.05, ** *p* < 0.01 and *** *p* < 0.001.

**Figure 8:**
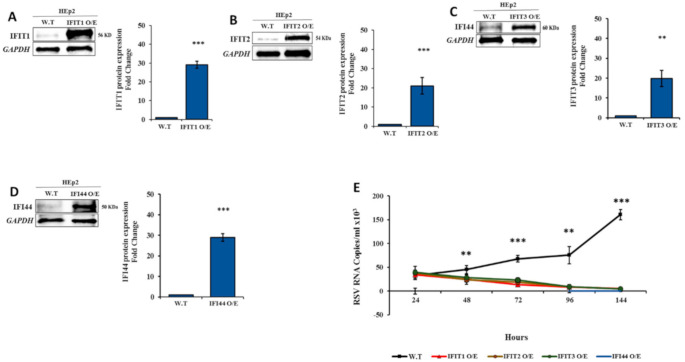
Infection of HEp2 -IFIT1/ IFIT2/ IFIT3/ IF44 overexpressing (O/E) cells with RSV. (**A**–**D**). HEp2 cells were transduced with a lentivirus system carrying IFIT1, IFIT2, IFIT3, or IFI44 pHAGE-DsRED(−)eGFP(+). Western blots analysis showing overexpression of IFIT1–3 and IFI44 expression (29.031%, 21%, 19.8%, and 24.20%, respectively) in infected as compared to untreated control cells. Error bars represent S.E. ** *p* < 0.01, *** *p* < 0.001. (**E**) IFIT1/ IFIT2/IFIT3/IF44- O/E HEp2 cells (1 × 10^6^) were infected with RSV (6 × 10^5^ PFU, 3 h). Supernatant was collected at predefined time points thereafter (24–144 h) and RNA was extracted. The control growth curve (black line) is RSV-infected HEp2 cells (W.T) and is the same data represented in Figure 2. qPCR analysis was performed to determine viral load. The data presented are an average of three independent replicates ± mean standard deviation. * *p* < 0.05, ** *p* < 0.01 and *** *p* < 0.001.

To assess the role of IFIT1–3 and IFI44 proteins in the inhibition of RSV infection, each gene was individually silenced/overexpressed in HEp2 cells. Silencing was confirmed by Western blotting (Figure 7A–D). Silencing of each of the four proteins separately resulted in increased RSV multiplicity as compared to wild type cells (*p* < 0.05) (Figure 7E). Overexpression of IFIT1-3 or IFI44 in HEp2 cells was confirmed by Western blotting (Figure 8A–D). Infection of HEp2 cells overexpressing *IFIT1–3* or *IFI44* individually with RSV, showed that the multiplicity rate of RSV was inhibited starting from 72 h post-infection (*p* < 0.01) (Figure 8E).

## 4. Discussion

In the current study, we noted an unusual overlapping peak of influenza and RSV virus activity in the 2016–20 winter seasons. This phenomenon is not unique to Israel, as a similar delay in RSV emergence was reported in Europe and was also observed with regard to other respiratory viruses, such as seasonal influenza and hMPV [23]. In vitro studies have reported that RSV and influenza share an ecologic niche [24], in which the growth of RSV is blocked by competitive infection with influenza [25].

Continual infection with influenza virus has been shown to lead to dysregulation of immune responses and hence to secondary infections [26,27]. In contrast, here we show, both in vitro and in vivo, that infection with influenza provided resistance to subsequent infection with RSV. More specifically, influenza infection upregulated anti-viral proteins that provided immunity against viruses. Indeed, most co-infections with influenza involve secondary bacterial infections and not viral infections [28,29].

The in vivo experiments presented here demonstrated that infection with influenza virus inhibits the ability of RSV to infect mouse lung cells. A two-wave modality of inhibition was noted, with increased RSV inhibition on days 2–5 and days 8–10 post-infection. Interestingly, the second wave of RSV inhibition occurred despite the absence of detectable influenza viruses. These waves were paralleled by a two-wave expression pattern of 10 proteins which have known antiviral activity, four of which belong to the IFIT family. We chose to focus on IFIT1–3 and IFI44 since it was shown that IFIT1–3 and IFI44 are key molecules in the antiviral pathway, where knockout of only one renders target cells more sensitive to viral attack [13,20]. More specifically, IFIT1 acts as a sensor that recognizes non-self RNAs, IFIT2 inhibits their translation or replication at the initiation stage [30], and IFIT3 acts as cofactor that stimulates IFIT1:2 complex stability and activity [31]. It was also shown that *Paramyxoviruses*, RSV among them, are strongly restricted in an IFITs-dependent manner, since they inhibit the translation of particular viral mRNA segments [32,33]. Furthermore, as IFIT1–3 and IFI44 were upregulated by influenza A, we hypothesized that they could be key players in RSV growth inhibition by influenza A.

The mechanisms underlying the two-wave expression of these 10 proteins are not fully understood. We demonstrated here that the second wave of protein expression occurred in the absence of influenza virus. In order to explain why a second wave of IFIT1–3 and IFI44 proteins expression occurred, RT-PCR analysis was conducted; our results suggest that this expression is likely the result of synthesis of new mRNA (Figure 6A,B). Interestingly, the same pattern of immune activity was observed two weeks after the inoculation of mice with the Sendai virus, when the virus was no longer detectable [34].

Acute viral infection induces alteration in the innate immune response, which then drives the development of chronic airway disease, which might then re-activate the innate immune system [35]. The presence of a persistent innate immune response on day 10 post influenza infection suggests that there is ongoing immune stimulation that might be mediated by viruses which are contained in macrophages or other cell types. It has been shown that co-infection of mice with RSV and influenza A viruses reduced disease severity compared to single infection (RSV or influenza A) [36]. RSV growth inhibition followed by influenza A infection may be explained by the fact that co-infection leads to downregulation of the RSV viral gene and significant upregulation of the influenza A polymerases PB1 and PB2 genes even seven days post influenza infection [37]. PB2 was found correlated with stimulation of natural killer T cells (NKT), which is known to lead to expression of interferons and stimulation of antigen presenting cells (APCs) associated with the clearance of viruses during infection [37]. For example, conventional dendritic cells, which are sites of virus uptake, are capable of activating NKT cells at low levels of antigen [38,39] and may stimulate anti-viral pathways, such as the ISGs pathway [40]. Future works must therefore focus on the IFIT1–3 and IFI44 proteins, as well as on other yet identified proteins that induce chronic innate immune activation after viral infection and on the possibility that viral remnants contained in macrophages or other cell types, drive this process.

## Figures and Tables

**Table 1:** Proteins demonstrating a two-wave behavior of upregulation in mouse lungs following influenza infection and are known to have antiviral activity.

Protein Names	Function
Eif2ak2	Inhibits viral replication via phosphorylation of the alpha subunit of eukaryotic initiation factor.
Gbp1	Promote oxidative killing and deliver antimicrobial peptides to autophagolysosomes.
Phf11	Inhibitor of prototype foamy virus (PFV) replication.
Ifi44	Exhibits an antiviral activity against hepatitis C virus.
Ifit1	Acting as a sensor of viral single- stranded RNAs and inhibiting expression of viral messenger RNAs.
Ifit2	Distinguish between self and non-self mRNAs by the host during viral infection.
Ifit3	IFN-induced antiviral protein which acts as an inhibitor of viral processes, and viral replication.
Ifitm3	Inhibits the entry of viruses to the host cell cytoplasm.
Oas3	dsRNA-activated antiviral enzyme which plays a critical role in cellular innate antiviral response.
Zbp1	Participates in the detection by the host’s innate immune system of DNA from viral.

## Supplementary Materials

The following are available online at https://www.mdpi.com/1999-4915/12/10/1171/s1, Figure S1: Co-infection of HEp2 cells with influenza A/H3N2 and RSV.
