# Supplementary material for: Influenza A Virus Inhibits RSV Infection via a Two-Wave Expression of IFIT Proteins
Source: Viruses. 2020 Oct 16;12(10):1171. doi: 10.3390/v12101171 (PMC7589235; doi:10.3390/v12101171)
Supplement: Supplementary file 1 [file viruses-12-01171-s001.pdf]

Supplementary

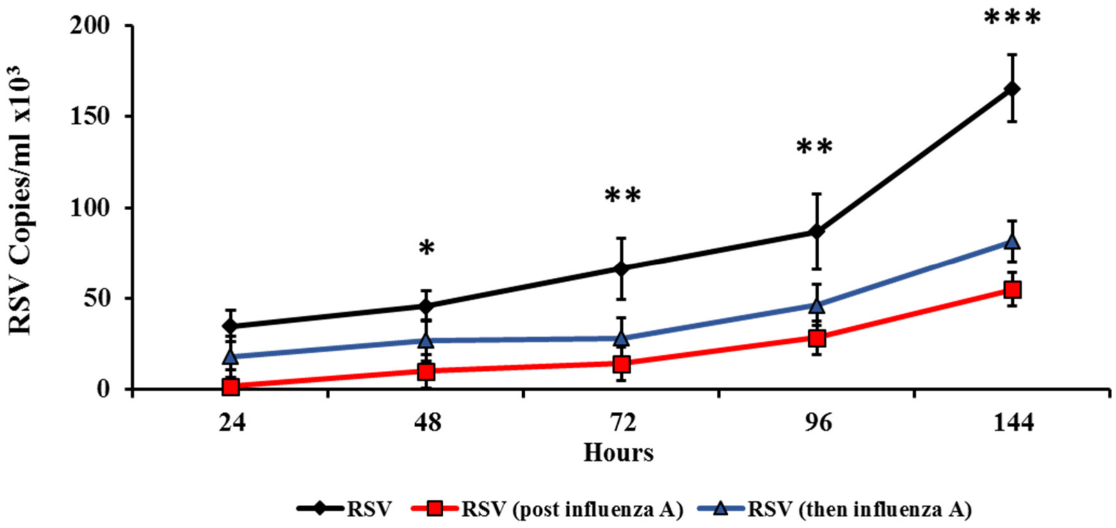

Figure S1. Co-infection of HEp2 cells with influenza A/H3N2 and RSV. HEp2 cells (1×10^6) were infected with influenza A/H3N2 (6×10^5 PFU) for 12 hours, and then with RSV (6×10^5 PFU), and vice versa. RSV-infected cells served as control. RNA was extracted from supernatant samples collected at predefined time points (24–144 h). qPCR was performed to test for viral quantity. The black line indicates infection with RSV, the red line indicates infection with influenza A/H3N2 followed by RSV, and the blue line indicates infection with RSV followed by influenza A/H3N2. The data presented is an average of three independent experiments ± mean standard deviation. *p <0.05, **p <0.01 and ***p <0.001.
